# In-Between Policy Vision and Practical Realities of Primary Healthcare: A Case Study in Rural Northern Sweden

**DOI:** 10.34172/ijhpm.8372

**Published:** 2024-11-18

**Authors:** Hanna Blåhed, Frida Jonsson, Anna-Karin Hurtig

**Affiliations:** ^1^Department of Epidemiology and Global Health, Umeå University, Umeå, Sweden.; ^2^Arctic Research Centre (Arcum), Umeå University, Umeå, Sweden.

**Keywords:** Rural Proofing, Integrated Care, Person-Centred Care, Good Quality, Local Healthcare, Sweden

## Abstract

**Background:**

In the context of a broader vision for primary healthcare (PHC) informed health systems, Sweden is following international trends by introducing the national "Good Quality and Local Health Care" reform. This reform seeks to establish a health system with primary care (PC) at the centre by emphasising aspects such as interorganisational collaboration and e-Health innovation. Since translating policy into practice may be challenging in rural areas due to resource constrains and normatively urban perspectives in national policy-making, this study explores how rural PC actors navigate the PHC vision in the context of a sparsely populated area of the Swedish north.

**Methods:**

This was a single case study, focusing on a rural municipality in northern Sweden. Thematic analysis was applied to data collected through interviews and observations, resulting in the development of three themes.

**Results:**

The results indicate that the policies were suboptimally aligned with the needs of the rural municipality. The results highlighted enduring collaborations that predated the reform. These local alliances led to a resource allocation challenge, rendering the existing networks and reform efforts concurrently understaffed. Moreover, the reform’s efforts to digitise healthcare faced impediments due to challenges associated with scaling up e-Health technology. Although key reform concepts such as person-centeredness and integrated care had already been put into practice, they were insufficiently acknowledged as such by external stakeholders.

**Conclusion:**

Subjecting national health policy-making to scrutiny by different stakeholders through the use of rural proofing can lead to a more deliberate and impactful implementation of policies. Rural proofing facilitates the pre-emptive identification of potential shortcomings, thereby enabling the formulation of necessary adjustments that resonate with local needs. This study shows apparent misalignments between the national vision and the practical reality in rural areas, therefore calling for greater efforts to include rural perspectives in national policy-making.

## Background

**Key Messages**

**Implications for policy makers**

- Policy-mak‎ers need to recognise the challenges posed by limited human and financial resources in the rural primary care (PC) setting and consider strategies to address these resource constraints for effective policy realisation. Adequate support for rural PC is crucial for aligning policy vision with the day-to-day work of healthcare providers.
- Policy-mak‎ers should learn from rural practices and procedures, recognising that solutions developed at the local level has the potential to positively impact national policy-mak‎ing.
- Policy-mak‎ers should be mindful that national policy-mak‎ing may be discouraging in local contexts. Consequently, the formulation of new practices and policies should consider the need for rural proofing to better align with and support local circumstances.

**Implications for the public**

Sweden’s national public health politic is rooted in an equity perspective, emphasising the provision of good and equal health to the entire population. This study sheds light on the unequal preconditions in rural areas for realising a national vision for a primary healthcare (PHC) informed health system. The rationale behind the study was to bridge the knowledge gap concerning rural primary care (PC), highlighting both challenges and possibilities, so that rural communities can be better served by national policies. This research give voice to rural PC organisations that persistently provide care to their communities, even as they struggle to navigate the realisation of national policies.

Even before the COVID-19 pandemic, many high-income countries (HICs) set out to strengthen their health systems through policy reforms.^1,2^ This development has been a partial response to challenges relating to access, quality, integration, and continuity of care,^3-7^ but also to workforce shortages^8,9^ combined with the rise of chronic diseases and complex comorbidities.^8^ While these challenges are apparent in urban environments, it is likely that efforts to reform health systems in rural areas are even more taxing. This follows largely from financial, epidemiological, and demographic changes such as depopulation, declining fertility rates, and a disproportionally older than average population, which leads to a scarcity of resources available for rural health systems development.^10-12^ In addition, many rural areas in HICs struggle with significant challenges in recruiting and retaining healthcare providers,^13-15^ which affects rural dwellers who tend to have less access to healthcare and yet experience poorer health outcomes compared to urban counterparts.^16-19^

 In response to these above challenges, which are present in urban settings but consequential in rural areas, the global movement towards more primary healthcare (PHC) informed health systems has gained particular prominence during last decades. Based on the Alma-Ata Declaration^20^ and further refined and reaffirmed in the 2018 Astana Declaration,^21^ PHC adopts a holistic approach to health and well-being, which includes health promotion and policy, as well as treatment and rehabilitation. The principles of PHC furthermore employ an inclusive approach, meaning that care shall be accessible and affordable in addition to comprehensive and coordinated. The related concept of primary care (PC) is placed within the framework of PHC, denoting essential, first contact care akin to a “family-doctor type service.”^22^ In order to implement the principles while addressing the challenges mentioned above, many HICs have started to transform their health systems towards a PHC informed service by emphasising aspects such as empowerment of individuals and communities, person-centeredness, and intersectoral collaboration.^23^

 Realising the visions of, and strategies in, PHC informed policies in rural contexts depends on similar conditions as in urban areas, such as having the skills and resources to do it.^24,25^ Yet, the conditions for successfully translating policy into practice sets rural and urban areas apart. National policies are typically formulated by policy-mak‎ers at the central decision-making level who, whether intentional or not, tend to adopt a normatively urban perspective.^26-30^ Furthermore, it is a common misconception that all rural areas are homogeneous, which can result in that “one-size-fits-all” policies become ineffective. To address this, rural proofing has been advocated by both the Organisation for Economic Co-operation and Development^31^ and the European Union (EU)-commission.^32^ This method involves examination of policies through a rural lens during both the policy-mak‎ing and implementation stages,^30,33^ thereby recognising diverse rural contexts. Policies that are likely to have differential impacts on rural areas should be adjusted accordingly.^33^ Specifically in the context of health policy-mak‎ing, rural proofing becomes crucial to ensure that health services effectively meet the needs of rural communities.^29,34^ Research has furthermore underscored the importance of involving stakeholders at various levels in the process of rural proofing, from politicians to healthcare providers and community members, as well as academia.^28^ The Swedish government have committed to carry out rural proofing in Sweden^35-37^ yet there is a deficiency of literature assessing the alignment of national policies with local contexts. This paper applies a rural lens to the implementation of health policies in the rural areas, exploring their congruence with local needs and priorities. To the best of our knowledge, few studies have documented the processes by which rural PC actors in Sweden navigate policies formulated at the national level.

 To contribute with knowledge on rural health policy implementation, a case study in Sweden was conducted with the aim of *exploring how the vision towards a PHC informed health system was navigated by actors in a sparsely populated area of the rural north.*

###  Reforming the Swedish Health System Towards Good Quality and Local Healthcare

 Sweden’s healthcare system operates within a framework of social protection, predominantly funded through taxation, and publicly provided. Provider payment mechanisms differ slightly among county councils. PC providers mainly receive capitation payments.^38^ The overarching goal is to ensure good health and care on equal terms for all citizens.^39^ However, the efficacy of PHC provision in Sweden has been a subject of ongoing debate since the establishment of the first primary health centre in 1968.^40^ Persistent challenges, such as prolonged waiting times^41^ and concerns about care quality (eg, in relation to elderly care, see Trydegård^42^), have consistently featured in Swedish health policy discussions. Despite numerous attempts to address these challenges, for example through introducing privatisation within the Swedish healthcare system in 2008, effectiveness-related issues persist, promoting further revisions, and adjustments in the healthcare system.^43^ Presently, efforts are directed toward transitioning to a health system informed by the principles of PHC as expressed in recent policy developments,^44-46^ most ardently articulated in the policy document “Good Quality and Local Health Care”from 2020 (In Swe.“God och nära vård”).^47^ Through it, the government seeks to establish a modern, sustainable, equitable, accessible, and effective health system with PC at the centre. This reform also sets three specific goals for improvements of the health system, which are to increase: (*i*) accessibility; (*ii*) patient participation and person-centredness; and (*iii*) continuity of care.^48^ The reform furthermore favours a structure built on new technologies, and innovations.^49^ At an organisational level, the government envisions a health system where the 21 county councils and 290 municipalities, responsible for PC as well as health promotion, collaborate more closely across administrative levels.^50^ This collaborative approach aims to counteract the concurrently fragmented delivery of care to achieve a more “seamless” one.^47,48^ Additional aspects of a PHC informed Swedish health system moves beyond New Public Management practices, towards more inclusive and responsive leadership approaches that better recognises the professional judgement of healthcare providers.^51^ This approach has been referred to as “trust-based management.” Up to this point, national evaluations of the “Good Quality and Local Health Care” reform have shown that the anticipated transformation has only made modest progress at the regional level.^52-54^ Research has shown that policy reforms often result in uneven levels of implementation across the country, with the county councils adopting different approaches to policy implementation based on the principle of local self-government which gives them the right to free self-determination.^26,55,56^ Similarly, findings have indicated that county councils with extensive rural and remote areas are faced with unequal challenges compared to their urban counterparts, which is suggested to be a result of health policies often being better suited for urban areas.^26,57^ In the current study, we seek to further explore these issues by studying the PC management in a rural community in northern Sweden, and how they realise the vision for a PHC informed health system in their setting.

## Methods

###  Case Study Research

 To address the aim of how the vision towards a PHC informed health system was navigated by actors in a sparsely populated area of the rural north, a single-case study was carried out. As described by Yin,^58^ case study is an investigative method that seeks to explore a phenomenon (the “case”) in its true environment (the “context”).

###  The Rural Northern Sweden: Introducing Southern Lapland 

 Northern Sweden covers nearly two thirds of the country’s land area and is populated by 12% of the population.^59^ Since most people live along the eastern coast, the western inland is left sparsely populated.^60^ The political discourse of northern Sweden has to an extent been built on an urban/rural dichotomy.^59,61^ While the Swedish south has been illustrated as modern and “inhabited by progressive, mobile, and creative people”^61^ (p. 3), the north has often been labelled as backward, and less developed. Playing the role as Sweden’s “weaker region,” the north has been characterised by low economic growth, high levels of unemployment and depopulation.^60^ It has furthermore often been described as a geographically and developmentally remote,^59^ with a harsh climate, long distances between communities, and a weak labour market.^62^ In reality, the region is culturally diverse with a heterogeneous population including youth, migrants and a strong presence of an Indigenous population, the Sámi.^61,63^ Not least, northern Sweden is of growing political and economic interest because of its leading position in the ongoing “green” industrial transformation.^64^

**Figure 1:**
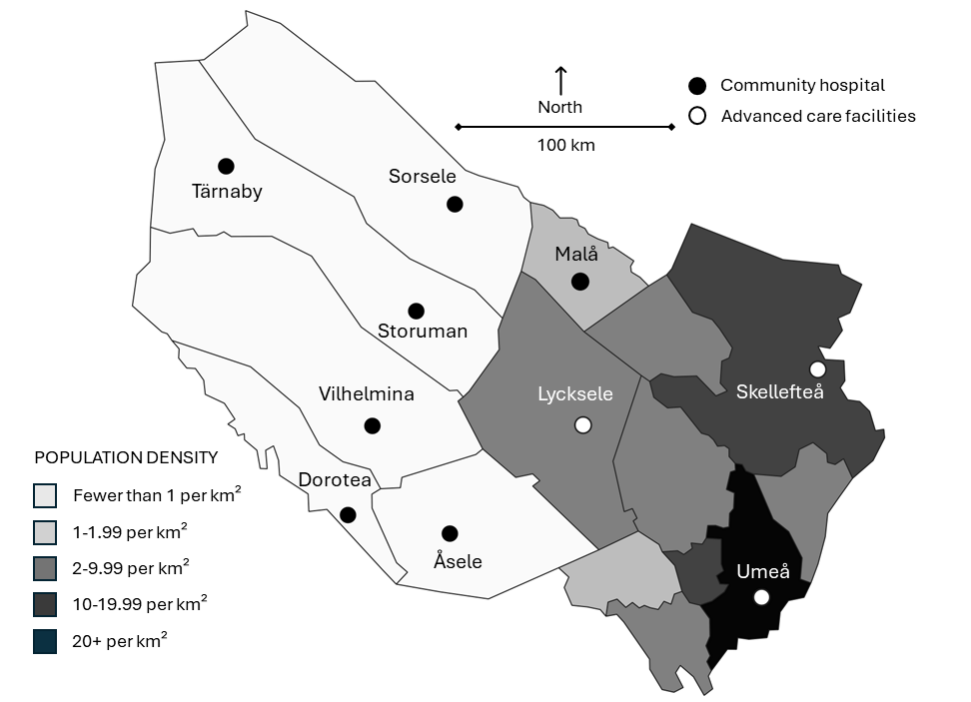
Illustration of Västerbotten County Including Southern Lapland.

 This study specifically concerns parts of northern Sweden called “southern Lapland” (Figure 1). During recent years, southern Lapland has been subjected to various innovation initiatives funded by the EU, the Nordic Council of Ministers, the Swedish Government, and Västerbotten county. These initiatives have had a broad focus on exploring approaches to economic and social development that could address the unique challenges posed by small and sparsely populated areas. Notably, in 2021, southern Lapland was chosen as a “model county” for Sweden’s “Good Quality and Local Health Care” reform^65^ which has allowed for increased governmental project funding. Being designated as a model county has led to the initiation of the flagship programme “Roadmap for service innovation,”^66^ which is a tool for implementing remote health and social services within the public sector. The launch of this program was strategically aimed at realising elements of the “Good Quality and Local Health Care” reform.

 Geographically, southern Lapland encompasses six municipalities that are classified as *rural* and *very sparsely populated,* meaning that their population resides exclusively in areas where the average travel time to a location with at least 50 000 residents is a minimum of 90 minutes.^67^ Nearly all municipalities in southern Lapland have a population where 30% is 65 years or older, which is ten percentages points above the national average. This indicates a high dependency ratio, signifying a substantial supply burden,^68^ which affects the predominantly publicly funded healthcare system, primarily funded through income-based taxes.^69^

**Figure 2:**
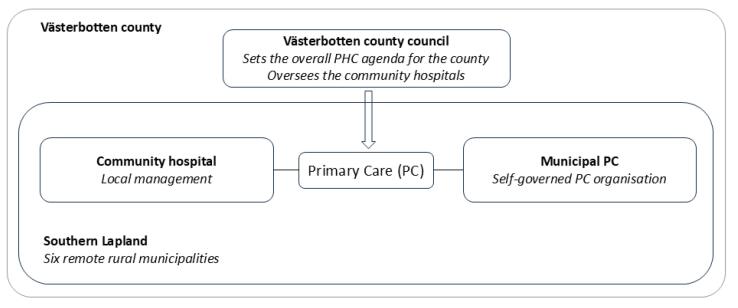
The Different Geographical and Administrative Levels in Västerbotten County. Abbreviations: PC, primary care; PHC, primary healthcare.

 In terms of healthcare provision in southern Lapland, Västerbotten county council oversees one hospital, two healthcare centres and six community hospitals in the area (Figure 2)^70^. The vast geographical distances make secondary care a restricted resource to many rural residents and the situation is compounded by limited options in terms of public transportation. The region’s older-than-average population results in the PC services, in terms of healthcare centres and community hospitals, dealing with intricate health issues more frequently than their urban counterparts.^71^ In the Swedish context, a community hospital is defined as a “hospital where the admission, care and discharge of patients are under the direct control of a general practitioner”^72^ (p. 92). Unlike a general health centre, they can offer a few hospital beds which allows for admission.

 In addition to healthcare services provided by the county council, the six rural and remote municipalities, acting as autonomous PC providers, bear the responsibility for financing, organising and delivering healthcare within ordinary and special housing for elderly people and people with functional impairments, as well as within in schools.^73^ Elderly care is particularly resource demanding for these municipalities since the number of home care patients surpasses the national average.^71^ Similar to the county council, the care offered by municipalities is mandated by the PC mission and a legal requirement, thus independent of the municipality’s size whether in terms of geography or population.^74^

###  Data Collection, Recruitment, Positionality, and Study Participants 

 In February 2023, the primary data collection was conducted through qualitative interviews with seven participants from one of the rural municipalities in southern Lapland. The cohort compromised two managers within the community hospital and two managers within the health and social care divisions at the municipality, as well as the head of the municipality. Additionally, a local part-time politician serving on the municipal board provided their perspective. Furthermore, an interview with a civil society representative was conducted to assess the role of community involvement in healthcare provision. This person was chosen due to their role in organising community stakeholders. The findings revealed that community engagement is not a required or common practice in rural healthcare provision.

 All three authors (HB, FJ, and AKH) are native Swedes with training and experience in public health and qualitative research. HB also has a background in political science, FJ in health promotion and evaluation, and AKH in medicine. They have previously conducted health systems research (HB, FJ, and AKH) and worked within the health system (HB and AKH) in rural northern Sweden. The three authors worked as a team and met regularly throughout the study to discuss and ensure that the study was guided by their expertise and their insider and outsider perspectives.

 The first author (HB) conducted the interviews, which were held in Swedish, using a thematic, semi-structured interview guide. They were held at the choice of the participant, predominantly in their professional space and were digitally recorded and transcribed verbatim in addition to the memos that were kept throughout the conversations. The interviews varied in length, ranging from 40 to 65 minutes.

**Figure 3:**
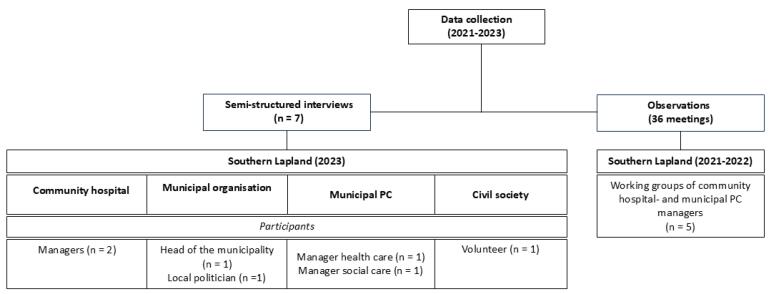
Overview of the Data Collection. Abbreviation: PC, primary care.

 The interviews followed and were informed by an observational study which proceeded from January-December 2022. Authors HB, FJ and AKH assumed the role of observers in the “Roadmap for service innovation” project (hereinafter referred to as the “innovation project”). It integrated the community hospitals and corresponding municipalities. We served as observers in five “working groups” (WGs) composed of managers from both health and social care divisions at the municipal level as well as managers from the community hospital. The project was overseen by a team employed by the county council. The proceedings of all WG meetings were conducted exclusively digitally. Our primary focus was on documenting the meeting procedure through notetaking. We took notes of the modus operandi among the WGs, how they interacted, operated within their context, and what results they produced. The report on the working process will be published separately, however we used the findings from the observational data to conduct context-specific interviews since they revealed crucial information of local processes that might otherwise have been overlooked.^75^ We attended 36 meetings spanning from 30 minutes to three hours (Figure 3).

###  Data Analysis

 Thematic analysis was applied to the interview data since it is a flexible analytical method, fitting to case studies. Guided by Braun and Clark, the six-step process was applied, which involved (*i*) familiarising with the data, including the transcriptions of verbal data; (*ii*) generating initial codes; (*iii*) searching for themes; (*iv*) reviewing themes; (*v*) defining and naming themes; and (*vi*) producing the report.^76^ Hence, the first author coded the transcripts, choosing codes that reflected the different ways that the vision towards a PHC informed health system was navigated by the rural PC actors (Table). The codes were shared and discussed with authors FJ and AKH and themes developed on account of codes being grouped. Led by the first author, preliminary themes were shared with the group and discussed. The observation notes were checked against the interview data to assess whether the two sources of data resonated with each other.^75^ To ensure the participants confidentiality, the notes are referred to only by date.

**Table:** Data Analysis

**Aim: exploring how the vision towards a PHC informed health system was navigate by actors in a sparsely populated area of the rural north Sweden**			
**Questions From the Interview Guide **	**Meaning Unit From Verbatim Transcript**	**Code From Meaning Unit **	**Theme**
[Q8] What opportunities exist for local customisation of healthcare reforms/policies within your organisation? Specifically, how can processes be adapted to align with your unique context?	*“We juggle a little bit here and there. There have been situations where you thought ‘this can’t be solved,’ but in some damn way we’ve solved it anyway, so flexibility and humanity and this that you do that little extra… that’s pretty much the culture we have, I would say.”*	Examples of person-centredness Local solutions to fit the context Adaptation Rural culture	Person-centeredness or just “rural health”?

Abbreviation: PHC, primary healthcare.

## Results

 Based on the thematic analysis, three themes developed: “Collaborative or constrained policy realisation,” “Digital dissonance,” and “Person-centredness or just ‘rural health’?” The first theme showcases successful, locallydeveloped collaborations, mainly between the community hospital and the municipality as envisioned in the policies. However, the theme also displays notable challenges that impede effective collaborations across administrative levels. The second theme, in turn, illustrates misalignments between the national vision and the practical reality in rural areas, manifested here in relation to digitalisation. The last theme captures concepts central to the national vision such as person-centredness, integrated care and trust-based management, while detailing how they are being realised in the rural context.

###  Collaborative or Constrained Policy Realisation

 The vision for a PHC informed health system in Sweden has been depicted in policies as a transformation essential to address existing and future challenges associated with demographic shifts and the growing healthcare demands of an ageing population.^57^ However, the evident need for change, as outlined in the policy documents and official statements^45,47,48^ seem to be challenged by the conditions of rural and remote municipalities. It was observed during the innovation project (note 2022-02-25) that in southern Lapland, the practical reality entailed a focus on addressing immediate issues, rather than working with long-term strategies. When inquired about navigating reforms like the “Good Quality and Local Health Care,” the head of the municipality described the situation by highlighting the diverse range of responsibilities delegated to municipalities, which is contingent on current politics and the global situation. This is illustrated in the following quote:

 “*These *[policies] *reflect the current situation. They *[the policies]* are delegated to the municipalities, but you get no resources for it, and that is probably the hardest thing. You have legal responsibilities, but you have no resources. And right now, the demands are numerous, we shall do this and this and this, but we do not get enough resources to do it. So then, you must do the impossible and find a level, that is good enough… we are a public sector, and we have statutory duties, so you just have to solve it, that’s it.*”

 The vision for a PHC informed health system furthermore strives towards a more comprehensive approach to care, through closer collaboration across organisational borders. However, as illustrated in the interviews, health governance in the rural and remote setting of southern Lapland has relied on collaboration long before the national vision was even formulated. Inter-municipal collaborative efforts, as well as partnerships between the municipality and the community hospital, have been imperative in the provision of welfare services such as PC, given the protracted constraints on both fiscal and human resources. As described by the head of the municipality, there are long-standing networks between municipalities, with one example being *Region 10 *which gathers the eight rural municipalities in Västerbotten county and two in Norrbotten county. Staffing the PC sector with skilled providers is a continuous challenge to the management, which requires collaboration across municipal, and even regional, borders. The head of the municipality highlighted the workforce challenges, stating:

 “*(...) the challenge of recruiting and retaining a skilled workforce exists throughout the county, and of course, it becomes particularly noticeable for inland municipalities. If we didn’t work together, it would never work.”*

 As illustrated in Figure 2, the municipality and the county council share the responsibility for PC but operate independently according to Sweden’s decentralised governance structure. The county council determines the overarching agenda for the county’s PHC which is within their mandate. Hence, healthcare decisions made at by the county council impacts not only the management of the community hospitals in the county, but also the municipal PC services. The interviews disclosed that agreements between the community hospital and the municipality could be contested and constrained by central decisions. A municipal PC manager recounted a recent incident where a local agreement between the municipality and the community hospital to ensure year-round access to their emergency ward was abruptly terminated due to a central decision by the county council. The municipal PC manager raised concerns about how this aligned with the vision of a PHC informed health system, by rhetorically asking “*To shut down the inpatient ward during the summer vacation, is it person-centred care?”*

 Although different forms of collaboration have long existed between the community hospital and the municipality, there appeared to be hardly any avenue for collaboration between the municipality and the county council, nor between the community hospital and the county council. The lack of structures for collaboration across administrative levels, particularly with the county council, was highlighted both in observations (note 2022-05-09) and interviews. When asked about this in one of the interviews, a manager from the community hospital reasoned as follows:

 “*I don’t really know what forum we have where we *[the community hospital and the county council] *can meet properly. We don’t have that kind of forum.”*

 As observed in meetings for the innovation project and throughout the interviews, structural limitations challenged collaboration across the administrative levels (note 2022-03-29). For instance, healthcare delivery is subject to several legislative contexts depending on the provider, either it be the county council or the municipality. As an example, patient records are documented in different journal systems, making it illicit for a nurse at the community hospital to share information to a caregiver employed by the municipality, in accordance with the Swedish Medical Act.^39^ The participants in the innovation project explained how patients discharged from inpatient care would transition to the care of the community hospital or municipal care without a comprehensive medical history (note 2022-05-07). Indeed, structural barriers were described by the participants as the main obstacle to achieve “seamless patient flow” as envisioned nationally. A manager at the community hospital went as far as stating that “*If the idea is that this *[provision of care]* shall run smoothly, then one should blur the borders and create something new*.”

###  Digital Dissonance 

 The vision for a PHC informed healthcare system in Sweden that is modern, sustainable, equitable, accessible, and effective includes a significant expansion of the digital infrastructure, with a particular focus on strengthening e-Health. Sweden’s overarching goal for e-Health is to become the “world’s best” in utilising digital technology in healthcare.^49^ As a result, various digital solutions are on the rise, encompassing more technologically oriented services like remote treatment, monitoring, and healthcare consultations.^77^ Against this backdrop, southern Lapland has become a testing ground for digitalisation and e-Health innovations, supported financially by the EU and the Swedish government. Several projects have been initiated and piloted, including the so-called virtual health rooms where digital technologies are used to enable community members to connect to distant healthcare providers.^78^ However, only a few of these innovations have transitioned into permanent solutions for everyday practice. This sentiment was echoed by a manager at the community hospital that remarked, “*here they talk about drones delivering medicine and all kinds of things but… then what?”*

 Some of the challenges encountered by the local PC services included inadequate support structures, notably the absence of information technology (IT) assistance and insufficient backing from the county council, which hindered the transition from project-level e-Health initiatives to their full-scale implementation (note 2022-10-31). According to the managers interviewed, there had been expectations from the national level of an expanded local implementation of e-Health; an aspiration that has not been properly matched with a consideration of necessary prerequisites. As illustrated in the quote below by a municipal PC manager, it was not clear to the management why digitalisation was taking so much time, or who would be responsible for scaling up pilots:

 “*Yes, yes progress is too slow. And that is what I’m wondering about, because the technology exists. ‘What is the problem?’ you wonder.”*

 While digital technology can improve access to care for rural and remote patients, in particular the frail or elderly, digital literacy is generally lower among the older population. It was depicted in the interviews that the burden of setting up or managing video consultations often fell short on the care providers. In southern Lapland, IT-support or similar services are limited, a reality that was further echoed by a manager at the community hospital who explained that *“we have no IT-support. We have no one, we have a small, small, IT-department that has absolutely no expertise regarding our stuff*.”

 Maintaining an existing digital infrastructure, or working to develop it, requires financial resources that the municipalities in southern Lapland do not have. In Sweden, municipalities can apply for grants via funders such as the EU or the government, but this administration requires resources that are lacking in rural municipalities overall, southern Lapland included. This situation evoked a frustration among municipal PC managers, with one of them explaining that *“it’s works for larger municipalities that have dedicated staff who just sit and work with *this [development grants and similar].* The rest of us get to do it with our left hand.”*

 Large-scale implementation of e-Health technologies and innovation has thus far proven challenging. However, the participants in the interviews explained that technical tools were currently being integrated, although not necessarily aligning with the vision presented for a PHC informed health system. For instance, the latest upgrade among the municipal healthcare providers, were the introduction of digital calendars in staff’s workstations. A municipal PC manager elaborated on this by describing:

 “*We’re trying to update our methods and go digital. Right now, the healthcare workers write their daily schedules on paper. We plan to start using the digital calendar in their work cells soon.”*

 One participant highlighted a particularly valued innovation that had been recently integrated: a distance spanning solution which allowed remote-working medical doctors to perform clinical rounds via video conference, supported by onsite nurses. The ability to avoid sending patients on long journeys for brief doctor’s appointments was described as a near-revolutionary advancement. Likewise, video calls were mentioned as useful in relation to home care visits by allowing municipal care staff to connect with the municipal nurse or the community hospital. A manager at the community hospital explained the benefits of video calls:

 “*The care giver can be seated on one side of the screen, while the patient is in a room here. We think that it is great, because sometimes … physical contact is not necessary for many consultations. It seems a bit redundant for patients to travel all the way to Umeå for such appointments *[indicating the county council hospital].”

###  Person-Centeredness or Just “Rural Health”?

 The vision for a PHC informed health system advocates for a shift towards person-centred approaches, which includes the concept of integrated care focusing on coordinated delivery.^5^ Continuity of care is also a concept deeply embedded in the health policy vision, concerning itself with the quality of care over time.^7^ The national vision has also emphasised the need for a shift in management, towards becoming more trust-based and less bureaucratic in terms of healthcare administration.^51^ The recognition of securing a local perspective in policy realisation is evident in the “Good Quality and Local Health Care” policy, among other current health policies.

 The interviews revealed that while certain local practices and procedures were not necessarily deemed or referred to as “person-centred,” concerns for community members were evident and seemed to encourage healthcare workers to discover effective solutions to meet patients’ needs. In other terms, person-centredness seemed to be imbedded in their way of work without being explicitly described as such, as illustrated by the below quote from a manager at the community hospital.

 “*We juggle a little bit here and there. There have been situations where you thought “this can’t be solved,” but in some damn way we’ve solved it anyway. Being flexible and showing compassion, and our ability to go the extra mile defines the culture we’ve cultivated here.”*

 Likewise, while it was never referred to as “integrated care” in the interviews, simplifying the care journey for community members was an evident priority. As illustrated in the quote below, the community hospital would assume responsibilities typically handled by the central specialist care, including arranging X-rays on-site, rather than referring patients to the county council hospital. This was told by one of the managers at the community hospital:

 “*Rather than requiring patients to make multiple trips to the county council hospital, we conduct X-rays here. We carry out follow-ups and investigations using blood samples which inevitably incurs costs.”*

 Despite the scarcity of healthcare staff in rural northern Sweden, one manager admittedly told that the continuity of staff was satisfactory and continued their argument by elaborating that maintaining a small yet consistent team improved supervision of patients, something that would not be possible in larger cities. The manager, which came from the municipal PC, said that *“so that’s the advantage, meaning, we keep it small, we can do good stuff.”*

 The concept of trust-based management is part of the vision for a PHC informed health system and known as a leadership approach in public management which has been initiated and encouraged by national policy-mak‎ers.^51^ The interviews demonstrated that the approach had been well-integrated to the municipal PC organisation, and it was explained partly by the long geographical distances between the management and the team of healthcare providers. The interviews underscored that managers’ trust in the staffs’ professional judgement was essential to secure a well-operating healthcare organisation across these vast areas. As the managers shared insights of their organisational environment, it became clear that together with their staff, they had operationalised the theoretical concept of trust-based management and turned it into an integral way of work. For instance, the managers trust in the staff had resulted in a shift in responsibilities and tasks related to scheduling, which a municipal PC manager shared as a positive experience:

 “*They decide a lot on their own and it works great that they do (…). They decide who should have vacation when, and *[they explain]* “here, we need more staff” and then it is me that have to find a temp. So, a lot goes out on them, because it becomes much better that it is they who do it *[scheduling]*. Everyone is happy, everyone gets the holiday they want.”*

 By fostering a working environment rooted in trust and collaboration between the management and the providers, local innovations were harnessed. A municipal PC manager recounted how different local needs were served by the PC team in a specific remote area of the municipality. The manager said:

 “*Our assistant nurses are willing to step in at the preschool when needed. For instance, if a staff member is unavailable for a short period, say an hour, my team has volunteered to assist. They proposed this idea, suggesting that when our patient schedule allows, they could supervise the children.”*

 In observations and interviews, it became clear that the community under study had developed its own capabilities to meet the PC mission and provide care. This meant being flexible, resilient, and innovative to meet the needs of the community. The head of the municipality simply concluded that “*there are not ten of us doing the same job, but one person doing ten jobs*.”

## Discussion

 The aim of the study was to explore how the vision towards a PHC informed health system was navigated by local actors in a sparsely populated area of rural northern Sweden. According to the policies of the reform, the vision of a PHC informed health system accentuates approaches to streamline healthcare delivery, for example, through collaboration across administrative levels, integrated care models, and new technologies.^44,46,47,49,51^ The findings from this study revealed a complex situation for local PC managers involved in health governance leaving them caught in-between the vision of national policies and the practical realities in their remote and rural area.^24,25^ As an example, if the policies in the “Good Quality and Local Health Care” reform had been evaluated with rural needs in mind, the gap between the lack of necessary e-Health infrastructure, such as sufficient IT support, and the goals of the vision could have been identified early on in the policy-mak‎ing process. Despite the prioritisation of broadband expansion on municipal and regional agendas across Sweden,^35^ the nation’s aspiration to become the “world’s best” in e-Health is hindered byinadequate IT support and challenges in scaling up e-Health technologies. On the other hand, findings from this study suggest that there are processes that could align effectively with the envisioned goals if they were duly acknowledged. The reigning political and public “discourse of decline”^79^ (p. 380) alludes to those areas faced with depopulation and slower growth as having has less capacity to adapt or transform in response to national demands. Nonetheless, the findings unveiled a far more complex reality in rural PC management. Indeed, southern Lapland has demonstrated a considerable amount of capacity, capabilities, and competences^24,25^ to fulfil their PC mission, aligning closely with the envisioned characteristics of a PHC informed health system. For one, the apparent smallness of the organisations in southern Lapland fostered a person-centred approach to healthcare delivery.^5^ The long distances between communities and healthcare teams resulted in a trust-based management approach between managers and healthcare providers.^51,80^ In terms of collaborative capabilities, southern Lapland has departed from the conventional narrative and, contrary to the common perception of the rural north, is at the forefront of many collaborative processes. Thus, contrary to popular beliefs, the supposedly “backward” rural north is in fact leading the way in several areas.^79^

 Sweden’s commitment to achieve equitable health outcomes across its areas necessitates strategic improvements in healthcare governance. By extending its scope, rural proofing strives to incorporate rural circumstances into daily policy-mak‎ing rather than treating them as separate from the broader government agenda, a process commonly referred to as “mainstreaming.” By learning from successful examples, such as England or Northern Ireland (where rural proofing is a mandatory practice), Sweden could aim for more inclusive policy-mak‎ing in regard to health policies. Currently, the implementation of rural proofing in Sweden appears inconsistent and fragmented.^36^ Although it is acknowledged by Swedish agencies as a useful tool^81,82^ it risks becoming an “empty gesture”^83^ (p. 114) or a mere ticklist exercise^33^ if not effectively applied. Indeed, as with many tools and practices, rural proofing is not without its methodological shortcomings. The prevailing approach is predominantly top-down, prompting scholars to advocate for the inclusion of local communities in policy assessment processes.^84^ What is more, concerns have been raised regarding rural proofing’s apparent oversight of diversity within rural contexts. Sherry and Shortall^30^ have persistently critiqued the prevailing urban/rural dichotomy, rather arguing that territorial space should be recognised in terms of their diversity, advocating for policies that are rooted in place-based realities. Saraceno posits that “the most important implication of the diversity concept as a way of understanding territorial differences is that policies need to be adapted and tailored to individual situations”^85^ (p. 336). She furthermore reiterates that top-down policies are less effective than bottom-up policies due to their one-size-fits-all approach. These perspective challenges the traditional defence of rural interests, which often relies on policies crafted around perceived needs. They suggest that the discourse should pivot to recognising the intrinsic value of rural areas, acknowledging their substantial contributions to the overall vitality, economic and social, of a region. Therefore, rural areas warrant targeted interventions aimed at bolstering the collective well-being.^30,86^

 Researchers have contributed with a variety of perspectives on the concept of rural proofing, converging on the consensus that regardless of the definitions of “rural,” policies must be attuned to the diverse needs of communities. Pertaining to healthcare policy-mak‎ing, there is a collective call for a comprehensive approach to rural proofing, necessitating the involvement of policy-mak‎ers, together with healthcare professionals in concert with the communities they serve. Additionally, the scholarly contribution, which advocates for interdisciplinary approaches, is imperative for a deepened empirical understanding of rural contexts and the formulation of well-informed policies. Indeed, research supports the fact that having a rural lens is essential to the future of rural healthcare.^28^

###  Strengths and Limitations of the Study

 As a research method, case study is continuously discussed in terms of its credibility, particularly regarding the generalisability of findings from single case studies.^87^ While this study’s limitation lies in being a single case study, the study sought to explore the phenomenon of translating a national policy vision into local practice within an under-researched context of the rural northern Sweden. The authors’ positionality as native Swedes with backgrounds in public health, political science and medicine may have influenced both data collection and analysis. Their familiarity with the Swedish health system and experience in rural northern Sweden provided valuable insights, but may also have led them to take certain norms and practices for granted, potentially overlooking alternative viewpoints. Previous experiences may have led them to emphasise findings that matched their expectations, and disciplinary backgrounds probably influenced the interpretation of the data. The use of different data sources, collected and analysed by all three authors, was a means of addressing these influences. Peer scrutiny in conferences and seminars also enhanced the research process and analysis. Conducting research in a small community presents challenges that indirectly impact research validity due to the limited number of potential participants. However, adhering to the principles of the Helsinki Declaration,^88^ the study’s strength lies in its focus on a rural setting and involvement of rural dwellers which is an often underrepresented group in research. In terms of transferability, the paper contributes a thick description of the study setting, offering a contextually rich narrative that enables readers to draw comparisons with their own situations.^89^

## Conclusion

 This study has highlighted both challenges and opportunities in realising the national vision for a PHC informed health system within a rural PC setting in northern Sweden. Drawing from an equity perspective, which underscores the extension of good and equal health to the entire population, this paper proposes strengthening the application of rural proofing, a lens that considers rural contexts, in policy-mak‎ing. The principal consideration in health policy may not lie in achieving equality between urban and rural areas, but rather in the pursuit of an equitable health system. Adopting a rural lens for policy recommendations could enhance the utilisation of existing local resources, innovations, and solutions, to fulfil their intended purpose more effectively. Similarly, this approach facilitates the identification of potential deficiencies within the prerequisites necessary for successful implementation of policy recommendations in the rural context.

 The body of academic research concerning rural proofing is notably scare, which positions this paper as a valuable contribution to the extant literature.^30,84^ This study applies a rural perspective to the implementation of health policy and shows that normative urban policy-mak‎ing hinders the enhancement and integration of existing local practices when these practices could in fact support the reform efforts. This paper furthermore offers valuable insights that can be applied to other HICs with extensive rural and remote areas facing similar challenges to their healthcare system. By aiming to narrow the knowledge gap related to health policy realisation in rural and remote areas in HICs, this study contributes to improving the understanding of how these areas navigate national health policies in their local context.

## Ethical issues

 The study has obtained ethical clearance from Swedish Ethical Review Authority (Dnr. 2019-01915). All participants received written information about the study beforehand and then gave their informed consent either orally or in writing to participate in the research. Data has been processed and stored according to General Data Protection Regulation (GDPR). The study took place in a small rural community and to ensure participants’ confidentiality, since there are few eligible participants and a significant degree of inter-organisational familiarity, the name of the municipality is not disclosed.

## Conflicts of interest

 Authors declare that they have no conflicts of interest.
